# Association of Microvasculature Changes with Visual Outcomes in Idiopathic Epiretinal Membrane Surgery: A Clinical Trial

**DOI:** 10.3390/jcm13164748

**Published:** 2024-08-13

**Authors:** Marie Henry, Ndeye Coumba Ndiaye, Karine Angioi-Duprez, Jean-Paul Berrod, Jean-Baptiste Conart

**Affiliations:** 1 Department of Ophthalmology, CHRU-Nancy, Université de Lorraine, F-54000 Nancy, France; k.angioi-duprez@chru-nancy.fr (K.A.-D.); jp.berrod@chru-nancy.fr (J.-P.B.); j.conart@chru-nancy.fr (J.-B.C.); 2 UMR Inserm U1256 NGERE (Nutrition-Genetics and Exposure to Environmental Risks), Université de Lorraine, F-54000 Nancy, France; ndeye-coumba.ndiaye@univ-lorraine.fr

**Keywords:** idiopathic epiretinal membrane, OCT-Angiography, retinal vessel density, best-corrected visual acuity

## Abstract

**Purpose:** The purpose of this paper is to evaluate macular microvascular changes and their correlation with visual outcomes after idiopathic epiretinal membrane (iERM) surgery. **Methods:** Forty-seven eyes operated for iERM were included in this retrospective case series. The foveal avascular zone (FAZ) area, and the vessel density (VD) in the superficial and the deep capillary plexus (SCP and DCP) were evaluated using optical coherence tomography angiography (OCTA). The association between the OCTA parameters and best-corrected visual acuity (BCVA) was examined preoperatively and postoperatively. Regression analyses were conducted to determine the potential predictive factors for visual recovery. **Results:** At baseline, the FAZ area in iERM eyes was significantly smaller than that in the control eyes (*p* < 0.001). iERM eyes also had a lower macular VD in both the SCP and the DCP (*p* < 0.001). Preoperative BCVA was negatively correlated with the FAZ area (r = −0.499, *p* < 0.001) and macular VD in the DCP (r = −0.422, *p* = 0.003). A negative correlation was also found between postoperative BCVA and macular VD in both the SCP (r = −0.394, *p* = 0.006) and the DCP (r = −0.569, *p* < 0.001). In the bivariate analyses, age, preoperative BCVA, iERM stage, and baseline macular VD in the SCP were significantly associated with BCVA at 6 months post-surgery. Multivariate regression analysis revealed that the preoperative BCVA was the only predictor of visual outcomes in iERM eyes (*p* < 0.001). **Conclusions:** Idiopathic epiretinal membrane (iERM) causes microvascular changes, including foveal avascular zone (FAZ) contraction and decreased macular vessel density (VD) in both the superficial capillary plexus (SCP) and the deep capillary plexus (DCP). These changes were significantly correlated with pre- and/or postoperative best-corrected visual acuity (BCVA). However, none of these alterations appeared to have prognostic value for visual outcomes in patients with iERM.

## 1. Introduction

Idiopathic epiretinal membrane (iERM) is a common retinal disorder characterized by fibrocellular proliferation over the internal limiting membrane [1]. The traction caused by iERM on the inner retina and overlying vessels may alter the morphology and the hemodynamics of the macula, leading to a variable loss of visual acuity and metamorphopsia [2,3].

iERM surgery has been an established technique for many years. It has a low complication rate, and functional improvement is seen in 70–80% of patients [4,5]. However, visual recovery may remain incomplete in some patients [5]. It, therefore, seems important to identify which markers can be predictive of post-operative visual acuity.

Some clinical factors have long been known, including age, duration of symptoms, and preoperative visual acuity [6,7]. The advent of spectral domain-optical coherence tomography (SD-OCT) has provided additional prognostic information, by highlighting the morphological changes associated with iERM [8]. Many previous studies have thus demonstrated that the presence of ectopic inner foveal layers, disorganization of retinal inner layers, and disruption of the ellipsoid zone and the interdigitation zone are significant predictors of poor visual improvement in iERM eyes [7,9,10,11]. However, a proportion of patients continue to present with visual impairment despite intact retinal layers, indicating the presence of microstructural damage that cannot be detected by SD-OCT [7,10,12].

Optical coherence tomography angiography (OCTA) is a non-invasive imaging technology that utilizes flow detection to achieve the detailed visualization of the macular microvasculature, encompassing the superficial capillary plexus (SCP), deep capillary plexus (DCP), and choriocapillaris plexus (CCP), without requiring dye injection [13]. It also has the potential to characterize the morphology of the foveal avascular zone (FAZ) and provide a quantitative analysis of the retinal and choroidal microvasculature with a high reproducibility [14].

Recent studies have provided evidence that iERM causes microvascular changes including FAZ contraction, increased foveal vessel density (VD), and decreased parafoveal VD [15,16,17,18,19,20,21,22,23,24]. Controversy still exists regarding the relationship between these variations and visual function improvement after iERM surgery. Some authors have shown a positive correlation with preoperative foveal VD in the SCP and DCP, while others have identified the superficial FAZ area and perimeter as potential biomarkers of postoperative visual outcome [15,18,21,25,26].

Conversely, another study showed no association between the OCTA parameters and visual acuity [16]. Therefore, it is unclear which OCTA features, if any, should be considered as prognostic factors for visual improvement in clinical practice.

The aim of this study was to investigate microvascular changes in the retina using OCTA and to identify potential predictive factors for postoperative visual acuity in patients with iERM.

## 2. Methods

### 2.1. Patients and Study Design

A single-center observational retrospective cohort study using medical record data was conducted on 140 successive patients who received surgery for iERM at the University Hospital of Nancy between November 2021 and February 2023. All patients were fully informed of the risks and benefits of the surgical intervention and gave their written consent before surgery. The study respected the tenets of the Declaration of Helsinki and the protocol was validated by the Ethics Committee of the Nancy hospital (n°2023PI177-433).

Inclusion criteria were as follows: (1) patients with visually significant iERM, and (2) a minimum follow-up period of 6 months after surgery. Exclusion criteria were as follows: (1) patients with iERM associated with vitreomacular traction (VMT) or macular hole, (2) patients with pre-existing macular pathologic features such as age-related macular degeneration or hereditary retinal dystrophies, (3) postoperative complications including cystoid macular edema and retinal detachment, and (4) unavailable, poor quality, and uncertain SD-OCT and/or OCTA images.

All patients underwent a comprehensive ophthalmological assessment at baseline, which included best-corrected visual acuity (BCVA) measurement using projected-light Snellen charts, axial length determination with the IOLMaster (Carl Zeiss Meditec AG, Jena, Germany), biomicroscopy with anterior segment evaluation, and fundus and macular imaging employing the Spectralis HRA-OCT (Heidelberg Engineering, Heidelberg, Germany) and spectral OCT RS-3000 Advance 2 + Angioscan (NIDEK, Gamagori, Japan). Patients were systematically evaluated within the first week post-surgery and, subsequently, at 1 and 6 months postoperatively. Each follow-up visit included a thorough ophthalmologic examination with macular imaging.

### 2.2. Surgical Technique

The same surgery was performed in all patients: a minimal three-port pars plana vitrectomy with 25-gauge instruments (EVA phacovitrectomy system, DORC, Zuidland, The Netherlands). After core vitrectomy, Brilliant Blue G (BBG) (ILM-Blue, DORC, Zuidland, The Netherlands) was injected over the posterior pole to evaluate the posterior hyaloid vitreous status and to stain the macula. If PVD was not already observed, the posterior hyaloid was elevated using either the vitreous probe or active suction through a blunt cannula. The edge of the iERM was then lifted with the microforceps. If no edge was observed, the membrane was grasped directly approximately 2 mm from the fovea and detached in a circular fashion. The internal limiting membrane (ILM) was then systematically searched for and, if necessary, deliberately removed after re-staining with BBG.

At the end of the procedure, the retinal periphery was meticulously inspected using a wide-angle viewing system (Oculus, Wetzlar, Germany) and scleral indentation. If a retinal tear was detected, vitrectomy was performed and the tear was treated with either laser photocoagulation or external cryoapplication.

Combined phaco-emulsification with posterior chamber intraocular implantation was applied in all patients over 60 years of age and in younger patients in case of cataract.

SD-OCT and OCTA imaging:

Microstructural imaging analysis of the fovea was performed using Spectralis HRA-OCT (Heidelberg Engineering Spectralis, Heidelberg, Germany). The SD-OCT protocol consisted of two high-resolution horizontal and vertical scans, ART5, of 6 mm and a volume scan using 60 µm equally spaced horizontal B-scans centered on the fovea and covering an area of 30° horizontally and 20° vertically.

Central macular thickness (CMT) was measured using the built-in Spectralis software.

The severity of the iERM was graded according to the OCT staging scheme proposed by Govetto et al. [9].

The NIDEK RS-3000 Advance 2 system with the latest AngioScan software (Version 1.8.0.14) was utilized to capture a 3 × 3 mm macular map centered on the fovea. Scans with a signal strength index below 7/10 were repeated. En face images of the retinal and choroidal layers were acquired following automatic layer segmentation and projection artifact correction using NIDEK OCTA software. All images were reviewed by a single investigator (MH) to confirm quality and consistency of the segmentation provided by the automated software. Any errors in segmentation were corrected, and images of insufficient quality were excluded.

**Figure 1:**
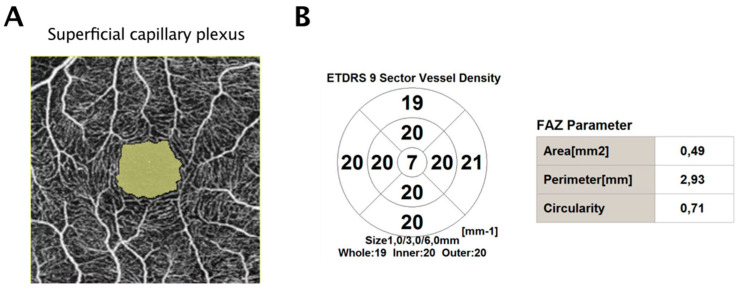
Quantitative measurement of optical coherence tomography angiography (OCTA) 3 × 3 mm scans in a normal eye. (**A**) Scan of the superficial capillary plexus. The FAZ area is depicted in green. (**B**) Sector vessel density and FAZ parameters. ETDRS: early treatment diabetic retinopathy; FAZ: foveal avascular zone.

The device’s analysis tool automatically computed the foveal avascular zone (FAZ) metrics of the superficial capillary plexus (SCP) and vessel density (VD) (measured in mm^−1^) for the SCP, deep capillary plexus (DCP), and choriocapillaris plexus (CCP) across the nine Early Treatment Diabetic Retinopathy Study (ETDRS) subfields. The ETDRS grid segments the macula into three concentric rings: the central 1 mm circle (fovea), the inner macular ring (parafovea), and the outer macular ring (perifovea) (Figure 1). We collected data on the FAZ area in the SCP and VD across the entire macular region, as well as the foveal, parafoveal, and perifoveal areas for both the SCP and DCP.

The ETDRS grid divides the macula into three concentric circles including the central 1 mm circle, the inner macular ring, and the outer macular ring which are referred to as the fovea, the parafovea, and the perifovea. The whole includes the central circle and the inner and outer rings.

### 2.3. Pre-, Intra-, and Postoperative Data

Pre- and intraoperative data included patient age and sex, axial length, lens status, BCVA, SD-OCT and OCTA findings, and type of surgery (single or combined procedure).

Postoperative data were BCVA and OCTA findings.

### 2.4. Main Outcome Measures

The evaluation of microvascular changes in iERM patients and the identification of potential prognostic factors for visual outcome were the primary endpoints of the study. The relationship between OCTA parameters and macular morphology and visual acuity before and after surgery was also investigated.

### 2.5. Statistical Analysis

Snellen visual acuity was converted to the logarithm of the minimum angle of resolution (logMAR) units for analysis. Categorical variables were expressed as numbers and percentages. Continuous variables were expressed as median and interquartile range, and the normality of their distribution was investigated using the Shapiro–Wilk test.

Comparison of OCTA parameters between operated and normal eyes and changes in OCTA parameters over time were investigated using the Wilcoxon signed-rank test. Spearman’s correlation coefficients were calculated to explore the association between OCTA values, and BCVA and macular morphology.

A bivariate linear regression was then used to estimate the relationship between the potential influencing preoperative factors and postoperative BCVA. Variables with *p* < 0.20 in bivariate linear regression were included in the final multivariate linear regression model.

The threshold for statistical significance was set at *p* < 0.05. Statistical analysis was performed using R version 4.2.1 (23 June 2022)

## 3. Results

During the study period, 140 eyes from 140 patients underwent surgery for idiopathic epiretinal membrane (iERM). Of these, 93 eyes were excluded for the following reasons: iERM associated with vitreomacular traction (VMT) and/or macular hole (*n* = 9), pre-existing macular pathology (*n* = 2), postoperative complications (*n* = 8), the absence or poor quality of optical coherence tomography angiography (OCTA) and/or spectral-domain OCT (SD-OCT) images (*n* = 46), and a follow-up duration of less than 6 months (*n* = 28). Consequently, 47 eyes from 47 patients met the study’s inclusion criteria.

### 3.1. Preoperative and Intraoperative Data

Baseline characteristics and intraoperative data are summarized in Table 1. Among the 47 patients, 18 (38.3%) were male and 29 (61.7%) were female, with a median age of 71.0 years [68.0; 76.0]. At diagnosis, 36 eyes (76.6%) were phakic and 11 eyes (23.4%) were pseudophakic, with a median axial length of 23.7 mm [23.0; 24.1]. The median preoperative best-corrected visual acuity (BCVA) was 0.3 logMAR [0.2; 0.4]. The distribution of iERM stages was as follows: 12 eyes (25.5%) had stage 2, 23 eyes (49%) had stage 3, and 12 eyes (25.5%) had stage 4.

### 3.2. OCTA Parameters in iERM Eyes and Comparison with Those of Fellow Eyes

**Figure 2:**
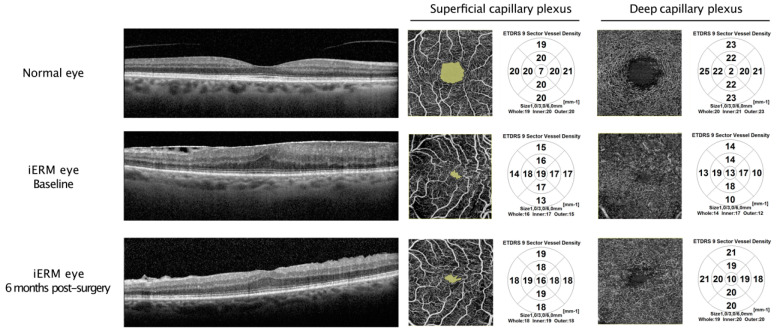
Representative SD-OCT and corresponding OCT-A images with ETDRS-based vessel density from the normal contralateral eye and the affected eye in a patient with iERM before and after surgery. ETDRS: early treatment diabetic retinopathy; iERM: idiopathic epiretinal membran; FAZ: foveal avascular zone; SCP: superficial capillary plexus; DCP: deep capillary plexus.

Table 2 presents the OCTA characteristics of eyes with idiopathic epiretinal membrane (iERM) at baseline and 6 months postoperatively, along with comparisons to the fellow control eyes. At baseline, the median foveal avascular zone (FAZ) area in iERM eyes was markedly smaller than in the control eyes (0.03 mm^2^ [0.03; 0.06] versus 0.3 mm^2^ [0.2; 0.3], *p* < 0.001). The median foveal VD was significantly increased while the median parafoveal and perifoveal VD were significantly decreased in both the SCP (*p* < 0.001, *p* < 0.001 and *p* < 0.001, respectively) and the DCP (*p* < 0.001, *p* < 0.001 and *p* < 0.001, respectively) compared with normal fellow eyes. Overall, iERM eyes had significantly lower macular VD in both the SCP and the DCP than control eyes (*p* < 0.001 and *p* < 0.001, respectively) (Figure 2).

All OCTA parameters in iERM eyes significantly improved over time. However, at 6 months, there were still significant differences between operated and control eyes in the FAZ area (*p* < 0.001), macular (*p* = 0.004), foveal (*p* < 0.001), parafoveal (*p* = 0.043), and perifoveal (*p* < 0.001) VD in the SCP and foveal VD in the DCP (*p* < 0.001) (Figure 2).

At baseline, the FAZ area (green) in the affected eye is significantly smaller than that in the normal contralateral eye. The whole VD in both the SCP and the DCP is also significantly decreased in the affected eye. After surgery, all OCTA parameters improved but did not reach the values of the normal fellow eye.

### 3.3. Correlation between OCT Parameters, Visual Acuity, and Macular Morphology in iERM Eyes

The median best-corrected visual acuity (BCVA) significantly improved from 0.3 logMAR [0.2; 0.4] at baseline to 0.05 logMAR [0.00; 0.19] at 6 months (*p* < 0.001). Preoperative logMAR BCVA exhibited a significant negative correlation with the baseline foveal avascular zone (FAZ) area (r = −0.499, *p* < 0.001) and with macular vessel density (VD) in the deep capillary plexus (DCP) (r = −0.422, *p* = 0.003). It also showed a trend towards a negative correlation with macular VD in the superficial capillary plexus (SCP) (r = −0.276, *p* = 0.059) (Table 3). A negative correlation was also found between postoperative logMAR BCVA and macular VD in both the SCP (r = −0.394, *p* = 0.006) and the DCP (r = −0.569, *p* < 0.001). (Table 3).

The median CMT significantly decreased from 480.0 [419.5; 525.5] µm at baseline to 391.0 [362.5; 416.0] µm at 6 months (*p* < 0.001). None of the OCTA parameters was correlated with CMT either preoperatively or postoperatively (Table 4). However, there was a positive correlation between iERM stage and macular VD in the SCP (r = 0.288, *p* = 0.049). (Table 4).

### 3.4. Predictive Factors for Postoperative Visual Outcomes

In the bivariate analyses, age (*p* = 0.026), preoperative BCVA (*p* < 0.001), iERM stage (*p* = 0.034), and baseline macular VD in the SCP (*p* = 0.042) were significantly associated with visual acuity at 6 months post-surgery (Table 5).

Multivariate regression analysis revealed that preoperative BCVA was the only predictor of visual outcomes in iERM eyes (*p* < 0.001)

## 4. Discussion

This study investigated microvasculature changes in iERM eyes and sought to determine whether these changes could serve as prognostic factors for visual outcomes following surgery.

Consistent with most previous reports, this series found that eyes with iERM had a smaller FAZ area, increased foveal VD, and decreased para- and peri-foveal VD, compared with normal fellow eyes [15,16,17,18,19,20,21,22,23,24]. These findings presumably reflect a macular contraction and a central vascular displacement caused by iERM, as Nelis et al. suggested [27]. Epiretinal membrane contraction may indeed exert anteroposterior and tangential tractional forces on the retina and its vasculature. Anteroposterior forces may cause vertical traction with macular thickening while tangential forces may drag the retina and displace the retinal vessels from the perifoveal to the foveal area. All OCTA parameters were found to improve after surgery, indicating that the retinal vessels may return to their original position with the release of iERM traction.

Numerous studies have investigated the impact of idiopathic epiretinal membrane (iERM) removal on microvascular changes, yielding contradictory results [17,18,19,20,22,23,24,28]. While some studies reported a significant reduction in foveal vessel density (VD) in one or both plexuses, others found no change compared to preoperative values [17,18,19]. Additionally, conflicting findings have been observed regarding changes in parafoveal VD and the foveal avascular zone (FAZ) area following surgery [17,18,19,20,22,23,24,28].

These discrepancies may be explained by differences in the OCTA device and examination, particularly the region of the macula that is analyzed. Various areas and subsectors of the superficial and deep macula were investigated, however, with different fields of scan (3 × 3 mm versus 6 × 6 mm) and defined boundaries, making a comparison across studies difficult. The follow-up period and time of assessment should also be taken into account when interpreting results. Told et al. observed significant changes in the FAZ area and superficial VD between the early postoperative period and 3 months post-surgery [19]. However, an improvement in OCTA metrics may also be expected beyond 3 months, as demonstrated by Mao et al. [18]. Finally, the disparate findings could be due to differences in inclusion criteria and iERM severity. Several authors have indeed shown that the degree of microvascular changes is strongly associated with iERM severity at baseline [17,19,20,21,25]. Furthermore, Mao et al., using a three-stage classification system, noted that OCTA parameters were more likely to improve in eyes with higher iERM grades [17]. It is, therefore, important to consider the iERM stage when evaluating postoperative OCTA changes following surgery.

In this series, all iERMs were graded according to the SD-OCT classification proposed by Govetto et al., which is mainly based on the appearance of inner retinal layers [9]. The macular VD in the SCP was significantly correlated with the iERM stage, indicating that the development of ectopic inner foveal layers is accompanied by the displacement of the superficial plexus. A similar trend was noted for the FAZ area; however, the result did not achieve statistical significance, likely due to the study’s limited sample size. This is in line with two previous series which reported that the FAZ area decreases with increasing iERM severity [21,25]. By contrast, Bacherini et al. reported that Govetto stages did not correlate with the VD in the SCP but rather with that in the DCP [25]. It is, however, reasonable to assume that iERM, which is located at the interface between the inner retina and the vitreous, primarily affects the SCP vessels and may cause a subsequent proportionate impairment in the DCP. The fact that the FAZ area and superficial macular VD did not recover after surgery but the deep VD did may also be indicative of greater damage to the SCP.

This study identified a correlation between several OCTA parameters and BCVA. At baseline, the FAZ area and macular VD in the DCP were significantly associated with BCVA while a trend toward significance was noted with the VD in the SCP. This is consistent with the study carried out by Bacherini et al. which reported, using the same OCTA device and examination protocol, that a smaller FAZ area, and lower whole VD in the SCP and the DCP were correlated to a lower visual acuity [25]. At 6 months, the correlation with the FAZ area disappeared but there was still a strong negative association between visual outcomes and macular VD in both the SCP and the DCP. One possible explanation is that vascular depletion may exacerbate the damage caused by iERM traction, resulting in irreversible macular dysfunction. Although the causal relationship remains to be elucidated, the reduced superficial VD in the macula, particularly in the parafoveal area, might also be indicative of neuronal damage secondary to mechanical distortion due to iERM. This assumption is based on Kim et al.’s study which found an association between a lower parafoveal VD in the SCP and lower parafoveal ganglion cell complex (GCC) thickness [28]. Because the thinning of the ganglion cell-inner plexiform layer after iERM surgery is thought to reflect the loss of retinal ganglion cells, they suggested that this association could be interpreted to mean that a lower parafoveal VD is related to GCC damage and subsequent worse visual outcomes [28,29]. Conversely, they found that a lower parafoveal VD in the DCP was associated with a thicker parafoveal inner nuclear layer (INL) both pre- and postoperatively [28]. It has been speculated that the DCP may contribute to the removal of excess fluid from the retina; its impairment could thus predispose the retina to accumulate fluid [30]. Yet, recent evidence indicates that the thickening of inner retinal layers including the INL is a good predictor of poor postoperative functional outcomes in patients with iERM [12,31]. The underlying mechanism is unclear but it is assumed that the disorganization of the INL and the accompanying cellular damage may impair the synaptic junction from the photoreceptor to ganglion cells, which may contribute to the visual deterioration in iERM eyes [31,32,33]. Together, these findings suggest that vascular damage in the DCP may cause visual loss through INL thickening. Another possible explanation is that the decrease in DCP vessels, which supply oxygen to the outer retina, may further deteriorate photoreceptor function, thereby compromising visual recovery.

There is a paucity of studies investigating the possible role of baseline OCTA parameters in predicting visual outcomes following iERM surgery [15,18,21,26]. Kim et al. identified FAZ parameters (area and/or perimeter) as predictors for postoperative visual acuity but they did not consider potential confounders in their analysis [15]. In this series, a lower preoperative BCVA, older age, and higher iERM grade were significantly associated with a worse visual recovery in the univariate model, confirming the findings of many previous studies [6,7,9,34]. The macular VD in the SCP but not the DCP was also found to be a predictive factor, which supports the idea that the SCP is the most affected and unlikely to recover despite effective iERM removal. However, in multivariate analysis, the preoperative BCVA was the only independent prognostic factor for visual outcomes. This is in line with a recent systematic review which reported that the baseline BCVA was the only variable consistently associated with postoperative BCVA in iERM eyes [7]. Although it is possible that macular VD in the SCP plays a role in predicting the BCVA in some aspect, its influence may not be as strong to compromise the effect of other factors, as evidenced by its close association with iERM grade and BCVA. These results stand in contrast with Feng et al.’s study which demonstrated that, after adjusting for confounding variables, preoperative foveal VD in the DCP was a valuable prognostic factor for iERM surgery [26]. Intriguingly, contrary to all previous reports, the baseline BCVA did not appear to influence visual outcomes in their multivariate model [26]. The causes for these discrepancies are unclear but could, again, be related to differences in inclusion criteria and OCTA device.

The limitations of this study include its retrospective design and relatively short follow-up period. It is possible that improvements in the best-corrected visual acuity (BCVA) may continue beyond 6 months post-surgery [35]. Additionally, eyes with severe retinal distortion and segmentation errors were excluded, potentially introducing selection bias. Baseline BCVA and OCTA measurements may have also been affected by the presence of cataracts in some patients; however, this potential bias is likely minimal, as images with a signal strength index below 7/10 were excluded.

In conclusion, this study demonstrates that idiopathic epiretinal membrane (iERM) induces microvascular alterations, including foveal avascular zone (FAZ) contraction and reduced macular vessel density (VD) in both the superficial and deep capillary plexuses. These changes were significantly correlated with pre- and/or postoperative BCVA, although none were found to be predictive of visual outcomes in iERM patients.

## Figures and Tables

**Table 1:** Baseline characteristics and intraoperative data for patients undergoing iERM surgery.

Baseline Data	Values
Number of eyes, n	47
Age, years (median (IQR))	71.0 (8.0)
Sex, *n* (%)	
- Male	18 (38.3)
- Female	29 (61.7)
Axial length, mm (median (IQR))	23.7 (1.1)
Preoperative lens status, *n* (%)	
- Phakic	36 (76.6)
- Pseudophakic	11 (23.4)
Preoperative BCVA, logMAR (median (IQR))	0.3 (0.2)
iERM grade, *n* (%)	
- Grade 1	0
- Grade 2	12 (25.5)
- Grade 3	23 (49)
- Grade 4	12 (25.5)
Central macular thickness, µm (median (IQR))	480.0 (106.0)
Surgical procedure, *n* (%)	
Combined cataract extraction	36 (75.6)

iERM: idiopathic epiretinal membrane; IQR: interquartile range; BCVA: best-corrected visual acuity; logMAR: logarithm of the minimum angle of resolution.

**Table 2:** Comparison of OCTA parameters between eyes who underwent iERM surgery and unaffected fellow eyes.

OCTA Parameters	Fellow Eyes	iERM Eyes		*p*-Value *		
		M0	M6	*p* ^a^	*p* ^b^	*p* ^c^
FAZ area, mm^2^	0.3(0.2)	0.03(0.03)	0.08(0.05)	<0.001	<0.001	<0.001
SCP, mm^2^						
Whole VD	18.0(1.5)	16.0(2.5)	18.0(1.0)	<0.001	<0.001	0.004
Foveal VD	8.0(2.0)	16.0(2.5)	13.0(3.0)	<0.001	<0.001	<0.001
Parafoveal VD	18.0(2.0)	16.0(3.0)	18.0(2.0)	<0.001	<0.001	0.043
Perifoveal VD	20.0(2.5)	16.0(2.5)	16.0(3.0)	<0.001	<0.001	<0.001
DCP, mm^2^						
Whole VD	17.0(6.0)	13.0(5.0)	17.0(3.5)	<0.001	<0.001	0.202
Foveal VD	5.0(2.0)	11.0(3.0)	7.0(2.5)	<0.001	<0.001	<0.001
Parafoveal VD	17.0(6.0)	14.0(6.0)	18.0(4.5)	<0.001	<0.001	0.402
Perifoveal VD	18.0(7.0)	13.0(6.0)	18.0(5.0)	<0.001	<0.010	0.709

iERM: idiopathic epiretinal membrane; FAZ: foveal avascular zone; VD: vessel density; SCP: superficial capillary plexus; DCP: deep capillary plexus * Wilcoxon signed-rank tests ^a^: iERM eyes at baseline vs. fellow eyes; ^b^: iERM eyes at 6 months versus baseline; ^c^: iERM eyes at 6 months vs. fellow eyes. Values are expressed as median with interquartile range.

**Table 3:** Pearson correlation analysis of OCTA parameters and pre- and postoperative BCVA in eyes who underwent iERM surgery.

Baseline Parameters	Preoperative logMAR BCVA		Postoperative logMAR BCVA	
	r	*p*-Value	r	*p*-Value
FAZ area, mm^2^	−0.499	<0.001	−0.059	0.689
SCP, mm^2^				
Whole VD	−0.276	0.059	−0.394	0.006
DCP, mm^2^				
Whole VD	−0.422	0.003	−0.569	<0.001

iERM: idiopathic epiretinal membrane; BCVA: best-corrected visual acuity; FAZ: foveal avascular zone; VD: vessel density; SCP: superficial capillary plexus; DCP: deep capillary plexus.

**Table 4:** Pearson correlation analysis of OCTA parameters and macular morphology at baseline in iERM eyes.

Baseline Parameters	iERM Stage		CMT	
	r	*p*-Value	r	*p*-Value
FAZ area, mm^2^	−0.267	0.069	−0.143	0.336
SCP, mm^2^				
Whole VD	0.288	0.049	0.164	0.270
DCP, mm^2^				
Whole VD	0.033	0.825	−0.138	0.352

iERM: idiopathic epiretinal membrane; CMT: central macular thickness; FAZ: foveal avascular zone; VD: vessel density; SCP: superficial capillary plexus; DCP: deep capillary plexus.

**Table 5:** Factors influencing visual outcomes after iERM surgery.

	Bivariate Analysis		Multivariate Analysis	
Factors	Beta Coefficient (Standard Deviation)	*p*	Beta Coefficient (Standard Deviation)	*p*
Preoperative BCVA	0.475 (0.052)	<0.001	0.457 (0.064)	<0.001
iERM stage	0.088 (0.040)	0.034	0.037 (0.029)	0.210
Age	0.008 (0.0038)	0.026	0.002 (0.003)	0.498
Axial length	−0.012 (0.019)	0.528		
CMT	0.0001 (0.0004)	0.675		
Vascular parameters				
Whole VD in the SCP	−0.024 (0.012)	0.042	0.008 (0.023)	0.728
Whole VD in the DCP	−0.013 (0.008)	0.095	0.005 (0.008)	0.550
FAZ area	−0.513 (0.393)	0.199	0.137 (0.153)	0.375

iERM: idiopathic epiretinal membrane; BCVA: best-corrected visual acuity; CMT: central macula thickness; FAZ: foveal avascular zone; VD: vessel density; SCP: superficial capillary plexus; DCP: deep capillary plexus.

## Data Availability

The protocol and the datasets generated during and/or analyzed during the current study are available from the corresponding author upon reasonable request.
